# Low Specificity but Dissimilar Mycorrhizal Communities Associating with Roots May Contribute to the Spatial Pattern of Four Co-Occurring *Habenaria* (Orchidaceae) Species

**DOI:** 10.3390/ijms24010665

**Published:** 2022-12-30

**Authors:** Wenliu Zhang, Jiangyun Gao, Shicheng Shao, Taiqiang Li

**Affiliations:** 1 Key Laboratory of Grassland Ecosystem of Ministry of Education, College of Grassland Science, Gansu Agricultural University, Lanzhou 730070, China; 2 Institute of Biodiversity, School of Ecology and Environmental Science, Yunnan University, Kunming 650500, China; 3 Gardening and Horticulture Department, Xishuangbanna Tropical Botanical Garden, Chinese Academy of Sciences, Mengla 666303, China

**Keywords:** *Habenaria*, orchid mycorrhizal fungi (OMF), endophytic fungi, fungi communities, network structure, co-occurring

## Abstract

Fungi with orchid roots have been increasingly proven to play important roles in orchid growth, spatial distribution, and coexistence of natural communities. Here, we used 454 amplicon pyrosequencing with two different primer combinations to investigate the spatial variations in the community of OMF and endophytic fungi associates within the roots of four co-occurring *Habenaria* species. The results showed that all investigated *Habenaria* species were generalists and the different fungi communities may contribute to the spatial separation of the four *Habenaria* species. Firstly, the fungal OTUs identified in the roots of the four species overlapped but their presence differed amongst species and numerous distinct OMF families were unique to each species. Second, NMDS clustering showed samples clustered together based on associated species and PERMANOVA analyses indicated that fungi communities in the roots differed significantly between the *Habenaria* species, both for all endophytic fungi communities and for OMF communities. Third, the network structure of epiphytic fungi was highly specialized and modular but demonstrated lowly connected and anti-nested properties. However, it calls for more soil nutrition and soil fungal communities’ studies to elucidate the contribution of habitat-specific adaptations in general and mycorrhizal divergence.

## 1. Introduction

Shifting fungal or pollinator partners in populations are believed to have been important for the diversification and the specialization of orchid species [1]. Orchids have an extreme dependency on compatible mycorrhizal fungi because germination of the dust-like seeds, almost lacking in nutritional reserves, and development of the heterotrophic protocorm require colonization by fungi providing organic carbon [2]. Most orchid species are thought to still be heavily reliant on mycorrhizal fungi for their mineral nutrition [3]. Owing to the critical reliance on symbioses for completion of orchid life cycles, differences in the mycorrhizal communities associating with orchids can be expected to mediate the abundance, spatial distribution, and coexistence of terrestrial orchids in natural communities.

In order to maintain spatial distribution and spatial isolation, it has been proposed that the diversity of symbiotic fungi in roots changes according to the orchid species and that different patterns can be present. Some studies believe that the specificity of mycorrhizal fungi in orchids is closely related to the orchid distribution [4]. For any given biotic interaction, a species can be a specialist if it associates with a single or a narrow range of partners, or a generalist if it associates with many or a broad range of partners. Many studies have shown that plants are often mycorrhizal generalists [5], in that they can interact with many taxonomically disparate mycorrhizal taxa. It is generally assumed that an orchid interacting with a broad range of partners may increase niche availability and allow survival in a large diversity of environments [6].

For the co-occurring orchid species, divergent mycorrhizal fungi associated between species can be expected to lead to small-scale habitat heterogeneity and reduced competition for resources [7,8,9,10,11,12]. It is commonly observed in orchids with highly spatially clustered distribution patterns that may have resulted from distance-dependent seed germination. Comparison studies on the spatial distribution of multiple orchids at a local scale have shown that species tend to be spatially segregated and associate with distinct sets of mycorrhizal fungi [7,8,9,10,11,12], suggesting that mycorrhizal fungi are important drivers of niche partitioning and contribute to orchid coexistence. However, it remains unclear to what extent differences in mycorrhizal communities contribute to spatial segregation and coexistence of orchid species, more comparison between species at difference scales are need to reveal the contribution to the co-occurring terrestrial orchids species.

So far, research about fungal diversity in orchid roots is mainly focused on the Orchid mycorrhizal fungi, which largely belong to the basidiomycetes and usually are members of the Tulasnellaceae, Ceratobasidiaceae and Serendipitaceae [5,13,14]. Moreover, some research has indicated that many orchid species, including photosynthetic orchids, simultaneously associate with a large diversity of ectomycorrhizal fungi belonging to the families Thelephoraceae, Inocybaceae and Tuberaceae [13,15,16,17,18]. Recently, the nonmycorrhizal fungi of orchids has also been studied due to their possible physiological functions, the synergistic or antagonistic relationships with other organisms they can provide for the advantages of orchids, and their potential as a source of bioactive compounds [8,19,20]. Particularly, an increasing number of studies have begun considering the fungi from both groups (mycorrhizal and nonmycorrhizal) in the network analysis to study the interactions between plants, microbes, and the environment among co-existing species. Therefore, knowledge about the different fungi associated with orchid roots can provide a better perspective on the interactions that occur in orchids’ natural habitats and how these fungi can contribute to orchid survival and adaptation. *Habenaria* Willd. is one of the largest genera of the orchid family and is widespread across the tropical and subtropical regions of the world [21]. *Habenaria* species mainly occur on rock outcrops, forest grass, thickets, and grasslands, and frequently have sympatric distribution [22]. Much of the research related to mycorrhizal associations with *Habenaria* species is aimed toward horticultural techniques, focusing on fungal species that initiate seed germination [23,24]. However, our knowledge of symbiotic fungi diversity associated with Habenaria and their contribution to spatial isolation between co-occurring Habenaria species is limited. There are 60 *Habenaria* species found in China [25], and several species often grow in the same place. In our field surveys on orchid species diversity in southwest Yunnan, we found that four *Habenaria* species, *H. davidii*, *H. fordii*, *H. petelotii* and *H. limprichtii*, are sympatrically distributed with overlapping flowering periods. To identify the potential contribution of the mycorrhizal fungi and the endophytic fungi associated with the roots to mediate the spatial distribution and coexistence of the four *Habenaria* in natural communities, we used 454 amplicon pyrosequencing to identify the fungi diversity and fungi communities in the four species and addressed the following questions: (1) Do these four species have a narrow or a broad OMF partner breadth at a local scale? (2) Does each orchid species share mycobionts or do they partition their niches by interacting with differences at the studied site? (3) Do the four species show differences in the network structures of endophytic fungi?

## 2. Results

### 2.1. Fungal OTUs

All 40 orchid individuals were colonized by mycobionts (proven by the existence of coils in the cortical root tissue) and PCR amplicons of all samples were obtained. The raw sequencing data generated by primer pair ITS3/ITS4OF and ITS86F/ITS4 in this study have been respectively deposited in NCBI SAR under the accession number SAR PRJNA866898 and SAR PRJNA866893. After quality-filtering and low-quality sequences, Illumina Miseq PE300 sequencing generated 1,844,402 (1175 OTUs) for ITS86F/ITS4 and 2,175,653 (1627 OTUs) for ITS3/ITS4OF, 1,715,590 (944 OTUs) and 1,642,501 (1456 OTUs) of which belonged to endophytic fungi (see Table S1). After analysis, 74.23% (870 OTUs) of the total number of sequences obtained for the primer set ITS86F/ITS4 and 73.04% (1332 OTUs) of the total number of sequences obtained for the primer set ITS3/ITS4OF could be assigned to Ascomycota and Basidiomycota. Rarefaction curves showed that the number of OTUs was relatively close to saturation for each individual plant (see Figure S1). 

According to Dearnaley et al. [4] and information from previous studies that detected mycorrhizal fungi from the roots and protocorm of *Habenaria* (see Table S2), 65 OTUs (76,218 sequences) for ITS3/ITS4OF and 39 OTUs (9234 sequences) for ITS86F/ITS4 were detected as potential orchid-associating mycorrhizal fungi. 

**Figure 1:**
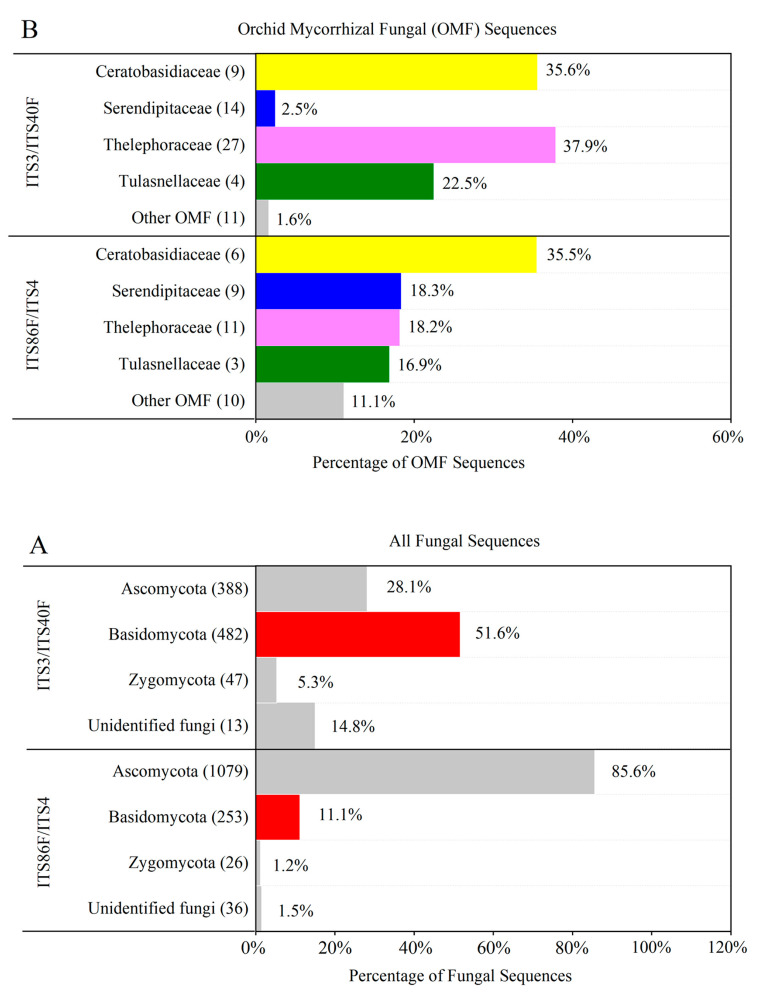
Frequency distribution of sequences generated from the four *Habenaria* root samples using primer combinations ITS3/ITS4OF and ITS86F/ITS4, including (**A**) all sequences identified by BLAST as representing fungal phyla, and (**B**) only those matching orchid mycorrhizal fungi (OMF) families previously described in the studied orchid species (refer to Table S2). Fungal phyla containing the most abundant OMF families are highlighted in red and the number of OTUs corresponding to each taxa are indicated within brackets.

The frequency distribution of sequences per fungal and subsequent OMF OTU was strongly dependent on primer combination (Figure 1). As expected, primer pair ITS3/ITS4OF showed a higher affinity for Basidiomycete phylum and ITS86F/ITS4 for the Ascomycete fungi (Figure 1A). Most Basidiomycete sequences obtained using ITS3/ITS4OF represented the families Thelephoraceae (37.9% of sequences identified as OMF; 27 OTUs) and Ceratobasidiaceae (35.6%; 9 OTUs). Similarly, the greatest number of sequences corresponding to OMF generated using ITS86F/ITS4 belonged to the Ceratobasidiaceae (35.5%; 6 OTUs) family, while OTUs corresponding to the other OMF were fewer in number for each primer set (Figure 1B).

### 2.2. OMF Diversity in the Roots of the Four Species

The fungi in the four main families (Ceratobasidiaceae, Tulasnellaceae, Serendipitaceae, and Thelephoraceae) can be found with a distinct OUT amount in the roots of each species by using ITS3/ITS4OF or ITS86F/ITS4. In terms of relative abundances of sequences, *H. davidii* and *H. fordii* both associate predominantly with the ectomycorrhizal taxa Thelephoraceae (84.3%, 14 OTUs and 83.9%, 19 OTUs), while the most abundant fungi detected in *H. petelotii* and *H. limprichtii* both belonged to the fungal family of Ceratobasidiaceae (68.6%, 3 OTUs and 29%, 4 OTUs) (see Figure S2A). For the primer sets ITS86F/ITS4, *H. davidii* associates predominantly with the ectomycorrhizal members of Serendipitaceae (59.3%, 5 OTUs), while *H. fordii* associates predominantly with the fungal family of Thelephoraceae (43.2%, 6 OTUs), but the most abundant fungi detected in *H. petelotii* were Ceratobasidiaceae (66.1%, 2 OTUs). In the roots of *H. limprichtii*, the member of Tulasnellaceae and Thelephoraceae was not detected and the other OMF, including fungal genus *Colletotrichum* (Glomerellaceae), *Tuber* (Tuberaceae), and *Phialocephala* (Vibrisseaceae), were the most abundantly detected fungi (see Figure S2B).

### 2.3. Shared Mycorrhizal Communities between Orchid Species

**Figure 2:**
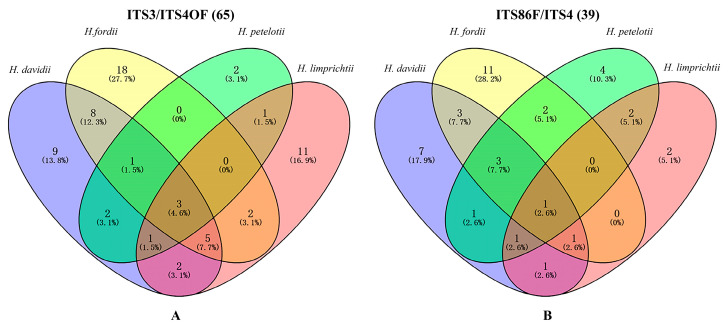
Venn diagrams showing the distribution of OTUs corresponding to OMF previously described in the studied orchid species (refer to Table S2) using primer pairs (**A**) ITS3/ITS4OF and (**B**) ITS86F/ITS4 between the studied orchid species (*Habenaria davidii*, *H. fordii*, *H. petelotii*, and *H. limprichtii*).

When comparing mycorrhizal fungal OTUs detected using ITS3/ITS4OF, there were 9, 18, 2 and 11 OTUs were included exclusively in the roots of *H. davidii*, *H. fordii*, *H. petelotii* and *H. limprichtii*, respectively (Figure 2A; Table S3). Although three shared OTUs were found in the roots of more than two species, the relative abundances of sequences were difference between species. The top three abundant fungi of each species were also different when comparing the relative abundances of all mycorrhizal fungal. Similar trends were observed with primer pair ITS86F/ITS4 and 9, 18, 2 and 11 OTUs were unique to H. davidii, H. fordii, H. petelotii and H. limprichtii respectively (Figure 2B, Table S3).

### 2.4. Differences in Endophytic Fungi and Mycorrhizal Communities between Orchid Species

**Figure 3:**
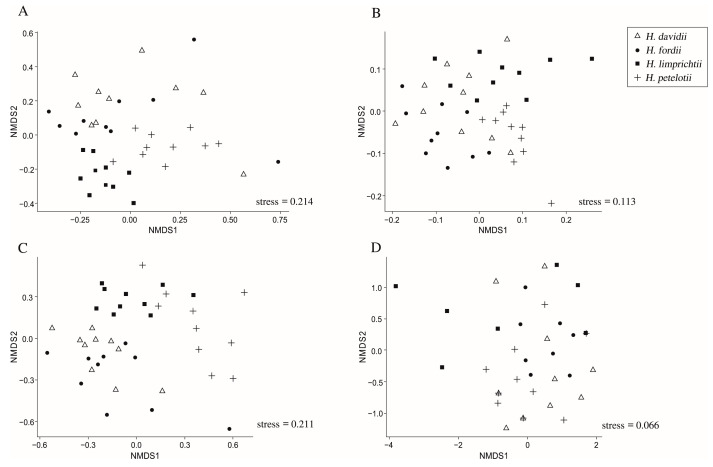
Non-metric multidimensional scaling (NMDS) plots based on all endophytic fungal OTUs and OMF OTUs obtained from the four studied *Habenaria* root samples using primer combinations ITS3/ITS4OF or ITS86F/ITS4 and clustered at 3% sequence dissimilarity. (**A**) All endophytic fungal OTUs obtained from ITS3/ITS4OF. (**B**) OMF OTUs obtained from ITS3/ITS4OF. (**C**) All endophytic fungal OTUs obtained from ITS86F/ITS4. (**D**) OMF OTUs obtained from ITS86F/ITS4.

NMDS clustering generated from all endophytic fungi found in the species showed similar trends in both primer pairs; samples clustered together based on associated species, with *H. davidii*, *H. petelotii*, and *H. limprichtii* roots clustered together but those of *H. davidii* scattered (Figure 3A,C). NMDS clustering generated from OMF OTUs produced by the primer ITS3/ITS4OF showed that *H. davidii*, *H. petelotii*, and *H. limprichtii* roots clustered together and those of *H. davidii* scattered (Figure 3B), while the OMF OTUs produced by the primer ITS86F/ITS4 showed a scattered arrangement (Figure 3D). However, the results of PERMANOVA analyses indicated that fungi communities in the roots differed significantly between the *Habenaria* species, both for all endophytic fungi communities (*p* < 0.001) and for OMF communities (*p* < 0.001), regardless of primer pair.

### 2.5. Network Structure of Plant-Endophytic Fungi with Primer ITSITS86F/ITS4 and Primer ITS3/ITS4OF

**Figure 4:**
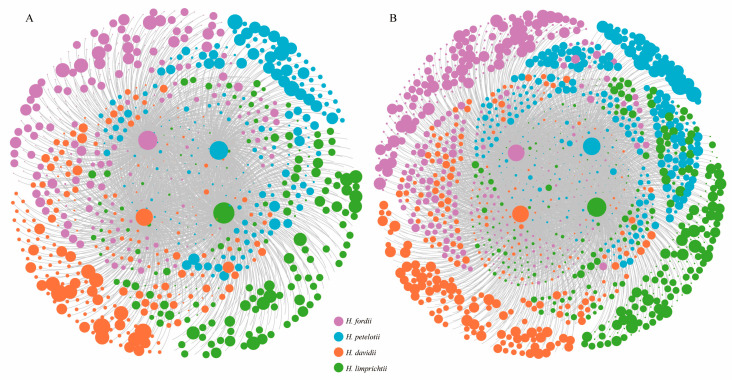
Visualization of the endophytic network. (**A**) ITS3/ITS4OF and (**B**) ITS86F/ITS4. The size of nodes roughly represents the relative abundance of fungal operational taxonomic units.

**Figure 5:**
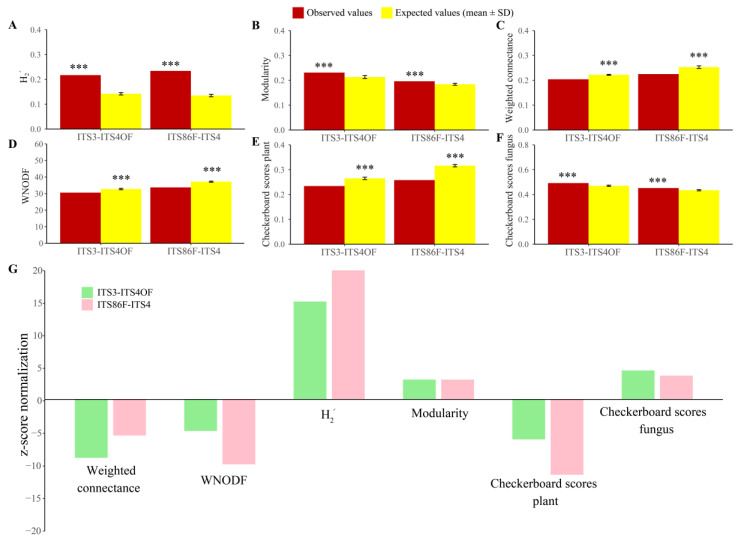
Architecture of the plant–fungus network. (**A**) H_2_′ metric of the network-level interaction specialization. (**B**) Barber’s metric of modularity. (**C**) Weighted connectance. (**D**) Weighted nestedness metric based on overlap and decreasing fill (WNODF). (**E**) Checkerboard scores representing the extent to which the overlap of fungi is avoided in the plant community. (**F**) Checkerboard scores representing the extent to which the overlap of plants is avoided in the fungal community. (**G**) Standardized network properties with Z-score normalization of endophytic networks from primer pairs ITS3/ITS4OF and ITS86F/ITS4. Asterisks indicate significant differences between the observed and expected values according to *t* test (*** *p* < 0.001).

The network of plants and epiphytic fungi with ITS86F/ITS4 and primer ITS3/ITS4OF is shown in Figure 4A,B. The network structure of epiphytic fungi both produced by ITS86F/ITS4 and by ITS3/ITS4OF was highly specialized and modular but demonstrated lowly connected and anti-nested properties. Particularly, the observed values of H_2_′ and modularity were significantly higher than expected based on null models (Figure 5A,B), whereas the observed values of the weighted connectance and the weighted nestedness metric based on overlap and decreasing fill (WNODF) were significantly lower than expected based on null models (Figure 5C,D). The observed values for plant and fungal communities shown by checkerboard score analysis was significantly higher than expected based on null models in both endophytic networks obtained from different primers (Figure 5E,F), indicating the existence of competitive interactions within the plant and fungal communities. Moreover, the Z-score normalization analysis indicated that the endophytic fungi produced by the primer ITS86F/ITS4 network was more highly specialized and more connected but similarly modular and more strongly anti-nested than those of primer ITS3/ITS4OF, and the degree of competitiveness within plant and fungal communities were greater in ITS86F/ITS4 producing endophytic fungi networks than in ITS3/ITS4OF producing endophytic fungi networks (Figure 5G).

## 3. Discussion

Our results show that the two different primer combinations, ITS3/ITS4OF and ITS86F/ITS4, generate a large number of fungal OTUs in our study, representing the broad fungal community numbers at the study location within the roots of the studied *Habenaria* species. Previous studies have shown that these two primer pairs were highly complementary and outperformed other primer pairs to characterize OMF communities [17,26]. Our studies revealed a large proportion of ascomycete fungi co-occuring with basidiomycete. These basidiomycetes were largely detected using the ITS3/ITS4OF primer pair, while ascomycetes were mainly detected using the ITS86F/ITS4 primer pair. This result is consistent with previous work that has researched the fungi communities of three cohabitating orchid species using this dual-primer combination [17].

By focusing only on the OTUs that have been described previously as putatively mycorrhizal in orchids, less than 100 different fungal OTUs were found in the roots of the investigated species. Previous studies of mycorrhizal fungi indicated that most terrestrial orchids associated predominantly with members of the Tulasnellaceae and Ceratobasidiaceae, while ectomycorrhizal taxa of the Thelephoraceae were present at low abundance [16,17,18,27,28]. In our study, *H. petelotii* and *H. limprichtii* mainly associated with members of the Ceratobasidiaceae when using the ITS3/ITS4OF primer pair. However, *H. davidii* and *H. fordii* associated predominately with Thelephoraceae. Simultaneously, the predominant mycobionts belonged to the ectomycorrhizal fungi (Serendipitaceae and Thelephoraceae) in the studied *Habenaria* species using ITS86F/ITS4, except those of *H. petelotii*. This pattern is consistent with previous work that has documented differences in mycorrhizal communities between three *Epipactis* species; the majority of mycorrhizal fungi of the three *Epipactis* species were ectomycorrhizal fungi belonging to Thelephoraceae, Serendipitaceae, and Inocybaceae [29]. Moreover, our results confirm earlier findings that members of Thelephoraceae have been shown to associate with *Habenaria radiata* [30], an exclusive *Habenaria* species that was studied about diversity of root-associated mycorrhizal fungi. In forest ecosystems, the mutually beneficial symbiotic relationship between ectomycorrhizal fungi and roots plays a key role in ecological functions and the mycelium can improve the plant’s resistance to drought and diseases [31,32]. The four studied Habenaria are all grown in sandy soil under forest shrubs and have high water requirements, which may partly explain why all the four species had a close relationship with those ectomycorrhizal fungi. Overall, our results demonstrated that the four studied orchid species associated with diverse ranges of mycorrhizal fungal symbionts simultaneously. Although it is not clear whether they are functional simultaneously, it is not unlikely that it optimizes access to plant growth-limiting resources, especially under nutrition-poor conditions. 

The surveys that have attempted to sample the large-scale distribution of mycorrhizal fungi associating with a particular orchid species have shown that the wide distribution of some orchid species may, to some extent, be explained by the widespread occurrence of their mycorrhizal associates [13,14,33,34]. *Habenaria* species have a widespread distribution across the tropical and subtropical regions of the world and occupy a variety of habitats types [21,35]. The four studied species widely distributed across Southeast Asia with various altitudes [36] and all studied species associated with several fungal OTUs simultaneously. Widespread orchids are often mycorrhizal generalists featuring flexibility in the OMF with which they associate at a geographical scale [37]. Our results showed that more than 10 OTUs were detected in each species, regardless of primer pair. Moreover, *H. fordii* and *H. limprichtii* associated with more than 25 fungal OTUs when using the ITS3/ITS4OF primer pair. This indicated that the four studied *Habenaria* species were generalists and associate with a wide variety of mycorrhizal fungal OTUs, although it remains unclear so far whether the different associate have a similar function towards the plant. Possibly, the ability to associate with several partners at the same time allows species to maximize their nutritional uptake. In most mature plants, growth is limited by either phosphorus or nitrogen, and it is reasonable to assume that mycorrhizal fungi expand the plant’s ability to forage these elements [38,39], particularly in places where nutrition is limited. Most *Habenaria* species typically occur in nutrition-poor habitats, such as open calcareous grasslands and/or forest edges with soil-covered rocks. Therefore, it might be hypothesized that increasing fungal breadth under nutrition-poor conditions may allow *Habenaria* species to maximize their nutrition uptake. However, more investigation about the soil nutrition of the four *Habenaria* species is needed in the further study. 

In addition to affecting growth and spatial distribution, the mycorrhizal communities are important drivers of niche partitioning for co-existing orchids. In our results, few fungal OTUs were shared between all studied species, with 3 OTUs detected using ITS3/ITS4OF and 1 OUT detected using ITS86F/ITS4. These shared OMF OTUs belonged to the Tulasnellaceae (ITS3/ITS4OF OUT 2530), Serendipitaceae (ITS3/ITS4OF OUT 4197), Psathyrellaceae (ITS3/ITS4OF OUT 8675), and Glomerellaceae (ITS86F/ITS4 OUT 4513). Although these “shared” OTUs were detected on all *Habenaria* species, they generally constituted a much higher proportion of sequences in one orchid. For instance, the sequence proportion of the share OUT 2530 was in 26.32% in *H. petelotii*, but that was much lower in *H. davidii* (4.68%) and *H. fordii* (3.48%), and the lowest was in *H. limprichtii* (0.07%) by using the primer pair ITS3/ITS4OF. Additionally, the dominated mycorrhizal fungi were distinct between species. For example, members of Thelephoraceae were the most dominant fungi associated with *H. davidii* and *H. fordii*, whereas members of Ceratobasidiaceae were most abundant in *H. petelotii* and *H. limprichtii* roots. Furthermore, although the dominant fungi of *H. davidii* and *H. fordii* both belong to the family Ceratobasidiaceae, the shared fungi between them were the members of the less abundant family. Despite the litter overlap in mycorrhizal partners, the NMDS and PERMANOVA analysis provided evidence for distinctive mycorrhizal communities associating with the four studied species (Figure 3). The different preferences for mycorrhizal fungi partners may promote coexistence by reducing competition for nutrients [40,41].

Theoretically, co-existent species do not compete for the same resources in natural ecosystems [42] unless small-scale habitat heterogeneity is present and therefore a partition of niches could be expected [43]. The pattern that differs the predominance of fungal partners among species in our study in consistet with previous findings that co-occurring orchid species tend to have distinctive mycorrhizal communities [10,12,17]. For example, Jacquemyn et al. [10] investigated the mycorrhizal community of seven co-occurring orchid species in Mediterranean grasslands, and found that co-occurring orchid species have distinctive mycorrhizal communities and show strong spatial segregation. Similarly, Waud et al. [17] showed that three co-occurring meadow species (*Orchis mascula*, *Anacamptis morio* and *Gymnadenia conopsea*) occupied different areas and associated with different mycorrhizal fungi. Whereas in the study by Jacquemyn et al. [29], the co-occurring, but distantly related, species *Epipactis neerlandica* and *E. palustris* had much more fungi in common with each other than with the *E. helleborine* that occurred in forests. It suggested that habitat variation plays a key role in mycorrhizal communities of terrestrial orchids. In our study, *H. davidii* and *H. petelotii* prefer open calcareous grasslands and forest, and *H. limprichtii* is mainly found in sparse grasslands on chalk and sandstone soil or forest under thickets, while *H. fordii* can be found in damp places or soil-covered rocks in forests or along valleys. *H. davidii* and *H. fordii* prefer to be associated with large numbers of ectomycorrhizal fungi of the Thelephoraceae family. The four species occupied various habitats with distinct fungi at a local-scale, which may contribute to species co-existence. Our results suggest that the pattern could result from differential performance of mycorrhizal fungi in sites with different habitat types. However, whether the composition of soil fungi and soil nutrition are various in this region, to what extent these differences are related to variation in fugal mycorrhizal communities in roots requires more evidence to clarify.

Network analysis showed that all endophytic fungi networks were characterized by high specialization and modularity, but there was low connectance and anti-nestedness in the results of both primer ITS3/ITS4OF and ITS86F/ITS4. This pattern is similar to previous orchid-mycorrhizal fungus networks [16,18,44,45]. In general, the orchid mycorrhizal network has significant characteristics of modules as a whole. For example, Martos et al. [42] built a binary network of nearly half of the tropical orchid species and 95 rhizoctonia fungi associated with them on Reunion Island, and found that the overall orchid mycorrhizal network showed high modularity due to the ecological barrier between epiphytic and terrestrial orchids. Similarly, Jacquemyn et al. [16] revealed that overall OMF diversity in a narrow transect of 10 × 1000 m with relatively similar habitats could be partitioned into a subset of 20 terrestrial orchids with mycorrhizal diversity belonging to five coexisting genera, low overlap among the subsets, and multiple isolated groups present in the interconnected network. However, some studies supported significantly nested network features [46,47,48]. In our study, although the four *Habenaria* orchid species are all simultaneously symbiotic with multiple endophytic fungi, the overall interconnected network remains highly modular due to the dominant OMF formed in the core of the network architecture. Notably, when considering the entire endophytic fungus, the symbiotic fungi community differences were the result of non-mycorrhizal fungi between studied *Habenaria* species to further expand and strengthen the differences in fungal populations, thus forming a very high modularity. Therefore, it is reasonable to speculate that the unique mycorrhizal fungi and the corn fungi group associated with the four species play a key role in their seed germination, seedling growth, or adult survival, and resulted in the distribution pattern and co-occurrence of the four Habenaria species.

## 4. Material and Methods

### 4.1. Study Species

All four studied species, *Habenaria davidii*, *H. fordii*, *H. petelotii*, and *H. limprichtii*, are terrestrial photosynthetic orchids that are widely distributed in southeast China, and can also be found in Southeast Asian countries with the exception of *H. fordii.* The species successively flower from July to August, and their fruits get mature in October. After the seeds are fully mature, the above-ground parts are completely withered, and only the tubers keep vitality. The new leaves usually emerge in the flowing spring. Previous studies on the four species indicated the difference of the floral period and floral morphology plays an important role in floral isolation among these four species [36]. However, the fungal associates of these four species in China remain unknown.

This study was conducted in Malipo, southeast Yunnan province of China. The four species were distributed along the roadsides from Daxiechang village (23°09′ N, 104°50′ E; alt. 1508 m) to Shangcuandong village (23°08′ N, 104°47′ E; alt. 2120 m), with about 19 km of road distance but only 7.3 km direct distance. *H. limprichtii* occurred in grasslands with calcareous soils and clustered at a higher altitude (>1800 m), while *H. petelotii and H. fordii* were sympatric and distributed in damp soil-covered rock places along valleys at a lower altitude (<1700 m). *H. davidii* can be found in open grassland or thickets at altitudes varying from 1700 to 1900 m. This region is a typical karst mountain landscape, and belongs to a subtropical plateau monsoon climate with average 1068 mm of annual rainfall and 17.6 °C of annual average temperature. 

### 4.2. Sampling

Samples were collected in August 2017 during a period when *H. petelotii* and *H. limprichtii* started flowering, but *H. davidii* and *H. fordii* were in the peaking flowering periods. For each species, ten individual plants were randomly selected and four root fragments (3–5 cm) were obtained from each individual (totally 40 samples). During sampling periods, we selected different orchid individuals that were at least one meters apart from each other to avoid the potential effects of disturbance and cloned individuals. Root samples were immediately placed into plastic bags after being surface cleaned several times with sterile water, and then were brought back to the laboratory with a 4 °C incubator on the same day. The roots were surface-sterilized in a 10% NaClO solution for 1 min and rinsed thoroughly with distilled water. Slight yellowish or opaque roots were selected and microscopically checked for mycorrhizal colonization. Roots were stored at −80 °C prior to molecular analyses of mycorrhizal associates. 

### 4.3. Molecular Analyses

For each sample, mycorrhizal roots were sectioned into 5–10 mm fragments and mixed, and then two separate 0.5 g root fragment subsamples were prepared for DNA extraction. Genomic DNA was extracted using a Ultraclean Plant DNA Isolation Kit as described by the manufacturer (Mo Bio Laboratories Inc., Carlsbad, CA, USA). To amplify the fungal internal transcribed spacer 2 (ITS2) regions, two complementary PCR primer combinations, ITS86F/ITS4 [49,50] and ITS3/ITS4OF [49,51], were used for polymerase chain reactions (PCR). PCR reactions were performed in triplicate 25 μL mixtures containing 5 μL of 5 × Pyrobest Buffer, 5 μL of 5 × GC buffer, 2 μL of 2.5 mM dNTPs, 1 μL of each primer (10 μM), 2 μL of template DNA, 8.75 μL ddH_2_O, and 0.25 μL of Q5 DNA Polymerase. The PCR program was as follows: 98 °C for 2 min, 25~30 cycles at 98 °C for 15 s, 55 °C for 30 s, and 72 °C for 30 s with a final extension of 72 °C for 5 min. Amplicons were extracted from 2% agarose gels and purified using the AxyPrep DNA Gel Extraction Kit (Axygen-Axygen Biotechnology (Hangzhou, China) Co., LTD) according to the manufacturer’s instructions and quantified using QuantiFluor^TM^-ST (Promega-GloMax Promega QuantiFluor). The purified amplicon mixture was subjected to high-throughput sequencing by Shanghai Personal Biotechnology Co, Ltd. using the Illumina Miseq PE300 sequencing platform (Illumina, Inc., San Diego, CA, USA), which generated 300 bp long paired-end reads.

### 4.4. Bioinformatic Analyses

Sequenced data was analyzed with the UPARSE algorithm implemented in USEARCH Ver. 8.0.1623 [52]. Paired-end reads of each sample were assembled into a single sequence and further filtered to discard reads when expected errors were more than 0.5. Assembled reads were trimmed at a fixed length of 230 bp and pooled where possible to maximize OTU discovery. During the process of OTU clustering, unique sequences (singletons), duplicated sequences, and chimeras were discarded. All generated OTUs were then supported by at least two assembled reads sharing a similarity of 97%, which is commonly used to delineate OTUs in fungal and orchid mycorrhiza communities. Taxonomy assignment for each OTU was performed using the UNITE database (http://unite.ut.ee, accessed on 21 September 2020) [53] through the PlutoF web workbench (https://plutof.ut.ee, accessed on 21 September 2020) [54]. To ensure the identity of mycorrhizal OTUs, we compared these OTUs against the NCBI nucleotide database (www.ncbi.nlm.nih.gov, accessed on 16 October 2021) using BLAST [55]. 

### 4.5. Data Analysis

In order to ensure the reliability and accuracy of the analysis, the OUTs with abundance <0.001% were removed [56]. The OTU identities sharing >90% sequence similarity with fungal species were considered as endophytic fungi. OTUs were then manually screened for possible orchid-associating mycorrhizal families based on the information provided by Dearnaley et al. [13] and previously detected mycorrhizal fungi from the roots, germinating seeds, and protocorms of various *Habenaria* species [23,24,30]. All endophytic fungi (both OMF and NOF) are reserved for subsequent overall and network analysis. Only OTUs that were detected on orchid roots and had a high BLAST identity (>90%) to known orchid-associating mycorrhizal families were retained for OMF analysis. 

After removal of OTUs identified as non-mycorrhizal, Venn diagrams showing the distribution of the OMF OTUs detected over the four orchid species were constructed from OTU tables generated by Venny 2.1.1 online (https://bioinfogp.cnb.csic.es/tools/venny/index.html, accessed on 15 January 2022). Distribution graphs were generated, ranking OTUs representing orchid-associating mycorrhizal fungi in order of declining number of sequences and partitioned according to their distribution between each sample type, further illustrating where these OTUs of interest were detected.

Non-metric multidimensional scaling (NMDS) plots clustering each individual root sample based on OTU presence absence were generated to visualize differences in fungal communities between orchid species using the vegan package for R [57]. To test whether endophytic fungi or mycorrhizal communities associating with the roots differed between the four orchid species, Permutational Multivariate Analysis of Variance (PERMANOVA, 999 permutations) using the adonis function in the vegan package [57] was conducted.

To visualize network structure for the plant endophytic fungi produced from primer ITSITS86F/ITS4 and primer ITS3/ITS4OF, networks based on the species-level matrices were explored and visualized using the interactive platform Gephi [58]. The architectural properties of the total endophytic networks based on the species-level matrices were then examined according to Guimerà and Amaral [59]. To perform a randomization test, randomized matrices were obtained based on the shuffle-sample null model with 1000 permutations. The network indices used in the analysis were the weighted connectance [60], the H_2_′ metric of network-level specialization [61], Barber’s metric of bipartite network modularity (Barber, 2007), the weighted nestedness metric based on overlap and decreasing fill (WNODF) [62], and checkerboard scores [63] representing the degree to which overlap of partners were avoided within the plant/fungus community. Calculations of the weighted connectance, H_2_′, WNODF, and checkerboard scores were performed based on the species-level original and randomized matrices using the network-level command in the bipartite package [64]. For modularity analysis, the species-level original and randomized matrices were binarized and output from R. Subsequently, the binary data were analyzed in the MODULAR program for simulated annealing-based estimation of network modularity [59]. Next, t tests were used to examine the differences between the observed and the random values at *p* < 0.05. To make comparisons between the two primers across networks, the network indices were standardized with Z-score normalization, which can correct for variation in species richness and the number of interactions [65]. The Z-score is defined as Z = (Eobserved − Erandomized)/SDrandomized), where Eobserved is the observed value and Erandomized and SDrandomized are the mean value and the standard deviation of the randomized matrices, respectively [65].

## Data Availability

The data presented in this study are available in article or Supplementary Material.

## Supplementary Materials

The following supporting information can be downloaded at: https://www.mdpi.com/article/10.3390/ijms24010665/s1.
