# Supplementary material for: Low Specificity but Dissimilar Mycorrhizal Communities Associating with Roots May Contribute to the Spatial Pattern of Four Co-Occurring Habenaria (Orchidaceae) Species
Source: Int J Mol Sci. 2022 Dec 30;24(1):665. doi: 10.3390/ijms24010665 (PMC9820590; doi:10.3390/ijms24010665)
Supplement: Supplementary file 1 [file ijms-24-00665-s001.zip › ijms-1967933-supplementary.pdf]

Table S1. Number of sequences and OTUs generated using UPARSE per primer pairs and percentage retained after manual screening of BLAST identifies.

	Total[a]		Confident fungal ID[b](Total endophytic fungi)		Mycorrhizal[c](OMF)		Unidentified	
	**Sequences**	**OTUs**	**Sequences**	**OTUs**	**Sequences**	**OTUs**	**Sequences**	**OTUs**
**ITS3/ITS4OF**	1844402	1175	1715590 (93.02%)	944(80.34%)	76218 (4.13%)	65 (5.53%)	127060 (6.89%)	217 (18.46%)
**ITS86F/ITS4**	2175653	1627	1642501 (75.49%)	1456 (89.49%)	9234 (0.42%)	39 (2.4%)	8424 (0.39%)	62 (3.81%)

[a]OTUs was calculated at 3% sequences dissimilarity and removed the low frequency OTUs with abundance < 0.001%

[b] OTU identities sharing > 90% sequence similarity with fungal species and which are based on sequence lengths >150 bp.

[c] Confident fungal OTUs with BLAST results matching fungal families described as OrM in the studied orchid species (Table S2).

Table S2. List of fungal taxa known or suspected to form orchid mycorrhizal (OMF associations with Orchidaceae species which was used to identify sequences corresponding to potential OMF. Analyses were restricted to those fungal taxa previously characterized in the studied species (*Habenaria* species) and other green terrestrial orchids, which are indicated in bold (adapted from Dearnaley *et al.,* 2012).

Phylum	Sub phylum	Class	Order	Family	Genus
Basidiomycota	Pucciniomycotina	Atractiellomycetes			
	Agaricomycotina	Agaricomycetes	Agaricales	Agaricaceae	Coprinus
				Inocybaceae	Inocybe
				Marasmiaceae	Gymnopus
					Marasmius
				Mycenaceae	Mycena
				Psathyrellaceae	Psathyrella
				Strophariaceae	Hymenogaster
			Cantharellales	**Ceratobasidiaceae**	Ceratobasidium
					Thanatephorus
				**Tulasnellaceae**	Tulasnella
			Sebacinales	**Serendipitaceae**	
			Thelephorales	**Thelephoraceae**	Thelephora
					Tomentella
			Russulales	Russulaceae	Russula
Ascomycota	Pezizomycotina	Pezizomycetes	Pezizales	Tuberaceae	Tuber
				Pezizaceae	Piziza
		Sordariomycetes	Incertaesedis	Glomerellaceae	Colletotrichum
		Leotiomycetes	Helotiales	Vibrisseaceae	Phialocephala

Table S3. Orchid mycorrhizal fungal (OMF) OTUs[a] detected by primer pairs ITS3/ITS4OF and IT86F/ITS4 and their frequency of association[b] with the different orchid species studies (*Habenaria davidii*, *Habenaria fordii*, *Habenaria petelotii* and *Habenaria limprichtii*).

	OUT identity based on Genbank match (Phylum-Family-OUT Nº)	Genbank Accession	*H. davidii*		*H. fordii*		*H. petelotii*		*H. limprichtii*		Sequences
			Individuals (X/10)	Association (%)	Individuals (X/10)	Association (%)	Individuals (X/10)	Association (%)	Individuals (X/10)	Association (%)	
ITS3/ITS4OF	Basidiomycota-Ceratobasidiaceae-Ceratobasidium-8465	WL1	1	0.77	1	0.02	4	99.21			11648
	Basidiomycota-Ceratobasidiaceae-Ceratobasidium-4479	2	1	0.13			3	99.87			11545
	Basidiomycota-Ceratobasidiaceae-Thanatephorus-6599	3			2	99.78			1	0.22	1359
	Basidiomycota-Ceratobasidiaceae-Ceratobasidium-1924	4			4	100					970
	Basidiomycota-Ceratobasidiaceae-Thanatephorus-863	5	1	100							20
	Basidiomycota-Ceratobasidiaceae-unidentified-245	6	3	73.39			1	0.07	2	26.53	1353
	Basidiomycota-Ceratobasidiaceae- unidentified -2051	7	1	94.78					1	5.22	134
	Basidiomycota-Ceratobasidiaceae- unidentified -7264	8							1	100	47
	Basidiomycota-Ceratobasidiaceae- unidentified -7786	9	1	100							27
	Basidiomycota-Tulasnellaceae-Tulasnella-3199	10	1	0.48					3	99.52	210
	Basidiomycota-Tulasnellaceae- unidentified -2530	11	3	7.59	3	8.01	9	84.39	2	0.01	10489
	Basidiomycota-Tulasnellaceae-unidentified-5460	12	2	99.86	1	0.02			1	0.12	6403
	Basidiomycota-Tulasnellaceae- unidentified -4881	13	1	100							20
	Basidiomycota-Serendipitaceae-unidentified-4197	14	2	4.94	1	0.31	4	67.77	2	26.98	971
	Basidiomycota-Serendipitaceae-unidentified-6321	15			3	100					203
	Basidiomycota-Serendipitaceae-unidentified-7878	16	1	32.14	2	67.86					168
	Basidiomycota-Serendipitaceae-unidentified-1821	17	1	99.22			1	0.78			128
	Basidiomycota-Serendipitaceae-unidentified-7937	18					2	99.19	1	0.81	123
	Basidiomycota-Serendipitaceae-unidentified-4329	19			2	100					75
	Basidiomycota-Serendipitaceae-unidentified-695	20					1	100			40
	Basidiomycota-Serendipitaceae-unidentified-2493	21							2	100	32
	Basidiomycota-Serendipitaceae-unidentified-2854	22							1	100	29
	Basidiomycota-Serendipitaceae-Sebacina-2511	23			1	100					28
	Basidiomycota-Serendipitaceae-unidentified-3531	24							1	100	25
	Basidiomycota-Serendipitaceae-unidentified-1249	25	1	100							24
	Basidiomycota-Serendipitaceae-unidentified-3434	26							1	100	23
	Basidiomycota-Serendipitaceae-unidentified-6031	27			1	100					20
	Basidiomycota-Thelephoraceae-Tomentella-1283	28	3	99.77	2	0.16			1	0.08	2565
	Basidiomycota-Thelephoraceae-Tomentella-1480	29			2	99.95			1	0.05	2189
	Basidiomycota-Thelephoraceae-Tomentella-4421	30	2	3.92	3	95.94			1	0.14	1430
	Basidiomycota-Thelephoraceae-Tomentella-3736	31	2	99.84	1	0.16					1282
	Basidiomycota-Thelephoraceae-Tomentella-2458	32	3	99.51	2	0.20			1	0.29	1017
	Basidiomycota-Thelephoraceae-Tomentella-2693	33					2	100			651
	Basidiomycota-Thelephoraceae-Thelephora-4471	34			5	100					369
	Basidiomycota-Thelephoraceae-Tomentella-8553	35	1	100							154
	Basidiomycota-Thelephoraceae-Tomentella-6842	36			1	100					147

	Basidiomycota-Thelephoraceae-Tomentella-2549	37			1	100					137
	Basidiomycota-Thelephoraceae-Tomentella-8633	38	1	100							135
	Basidiomycota-Thelephoraceae-Tomentella-3279	39	1	97.94	2	2.06					97
	Basidiomycota-Thelephoraceae-Tomentella-4761	40	1	100							91
	Basidiomycota-Thelephoraceae-Tomentella-2421	41							2	100	79
	Basidiomycota-Thelephoraceae-Tomentella-3248	42			1	100					54
	Basidiomycota-Thelephoraceae-Thelephora-6837	43			1	100					34
	Basidiomycota-Thelephoraceae-Tomentella-8162	44			1	100					33
	Basidiomycota-Thelephoraceae-Tomentella-6389	45	1	94.74	1	5.26					19
	Basidiomycota-Thelephoraceae-Tomentella-8358	46	3	0.15	6	99.78			1	0.07	14851
	Basidiomycota-Thelephoraceae-Tomentella-495	47	2	72.23	2	27.77					1271
	Basidiomycota-Thelephoraceae-Tomentella-5096	48	3	99.91	1	0.09					1127
	Basidiomycota-Thelephoraceae-Tomentella-3226	49			2	100					699
	Basidiomycota-Thelephoraceae-Tomentella-255	50	2	100							238
	Basidiomycota-Thelephoraceae-Tomentella-4028	51	1	98.53	1	1.47					68
	Basidiomycota-Thelephoraceae-Tomentella-739	52			2	100					58
	Basidiomycota-Thelephoraceae-Tomentella-6996	53							2	100	32
	Basidiomycota-Thelephoraceae-Tomentella-6038	54							1	100	27
	Basidiomycota- Agaricaceae-Coprinus-7218	55							1	100	32
	Basidiomycota- Agaricaceae-Coprinus-6035	56			1	100					104
	Basidiomycota- Marasmiaceae-Gymnopus-5289	57			1	100					35
	Basidiomycota- Marasmiaceae-Gymnopus-5100	58			1	100					32
	Basidiomycota- Marasmiaceae- Marasmius-3466	59	1	96.97	1	3.03					33
	Basidiomycota-Mycenaceae-Mycena-8108	60			1	100					39
	Basidiomycota-Mycenaceae-Mycena-7472	61	1	100							43
	Basidiomycota-Mycenaceae-Mycena-2057	62			1	100					59
	Basidiomycota-Psathyrellaceae-Psathyrella-8675	63	1	51.31	1	0.38	3	27.53	5	20.78	799
	Basidiomycota-Psathyrellaceae-Psathyrella-3549	64							1	100	41
	Ascomycota-Pezizaceae- Piziza-4253	65							1	100	33
	Basidiomycota-Ceratobasidiaceae-Ceratobasidium-1562						2	100			1484
	Basidiomycota-Ceratobasidiaceae-Ceratobasidium-3709						1	100			992
	Basidiomycota-Ceratobasidiaceae-Thanatephorus-6955				2	100					505
	Basidiomycota-Ceratobasidiaceae-Ceratobasidium-12552				3	100					163
	Basidiomycota-Ceratobasidiaceae-Ceratobasidium-11760				1	100					25
	Basidiomycota-Ceratobasidiaceae- unidentified -6053		2	17.59	1	0.93			3	81.48	108
	Basidiomycota-Tulasnellaceae-Tulasnella-6746		3	1.22	2	12.15	7	86.63	1		905
	Basidiomycota-Tulasnellaceae- unidentified -1207				3	50.28	2	49.72	1		354
	Basidiomycota-Tulasnellaceae-unidentified-4248		1	99	3	1.00		0.00			299
	Basidiomycota-Serendipitaceae-Sebacina-5306		3	99.9	1	0.10		0.00			1024
	Basidiomycota-Serendipitaceae-Sebacina-12935		1	84.43	1	0.55	1	15.03			366
	Basidiomycota-Serendipitaceae-unidentified-6882						1	20.78	3	79.22	77
ITS86F/ITS4	Basidiomycota-Serendipitaceae-unidentified-5070		1	22.95			1	52.46	1	24.59	61

Basidiomycota-Serendipitaceae-unidentified-10201				2	100					41
Basidiomycota-Serendipitaceae-unidentified-10954								2	100	37
Basidiomycota-Serendipitaceae-unidentified-4695		1	100							32
Basidiomycota-Serendipitaceae-Sebacina-4876		1	100							30
Basidiomycota-Serendipitaceae-unidentified-9680				1	100					26
Basidiomycota-Thelephoraceae-Thelephora-12406				2	100					189
Basidiomycota-Thelephoraceae-Tomentella-3842		1	100							157
Basidiomycota-Thelephoraceae-Tomentella-2198		1	100							121
Basidiomycota-Thelephoraceae-Tomentella-1974		2	100							113
Basidiomycota-Thelephoraceae-Tomentella-3422				1	100					86
Basidiomycota-Thelephoraceae-Tomentella-7071				2	100					70
Basidiomycota-Thelephoraceae-Tomentella-5401						1	100			65
Basidiomycota-Thelephoraceae-Tomentella-4663		1	30.77	1	69.23					26
Basidiomycota-Thelephoraceae-Tomentella-1649				1	100					25
Basidiomycota-Thelephoraceae-unidentified-6337				3	100					770
Basidiomycota-Thelephoraceae-unidentified-4186		1	100							56
Ascomycota-Vibrisseaceae-Phialocephala-10782								1	100	25
Ascomycota-Vibrisseaceae-Phialocephala-13239		3	50	1	2	1	48			50
Ascomycota -Tuberaceae-Tuber-4880		1	60.29					1	39.71	68
Ascomycota -Tuberaceae-Tuber-1233				3	100					206
Glomerellaceae-Colletotrichum-4513		2	13.65	4	83.76	1	0.37	1	2.21	271
Glomerellaceae-Colletotrichum-8833		3	34.29			2	65.71			70
Glomerellaceae-Colletotrichum-12073						1	13.2	1	86.8	197
Basidiomycota-Inocybaceae-Inocybe-5181				2	85.37	1	14.63			41
Basidiomycota-Mycenaceae-Mycena-884		4	100							60
Basidiomycota-Russulaceae-Russula-11282						1	100			39

[a] OTUs were calculated at 3% sequence dissimilarity. Only OTUs representing fungal taxa described as OMF in the studied orchid species (Table S2) were included.

[b] OUT frequencies of association% = The sequence of an OUT in a species/Total sequences of the OUT*100.

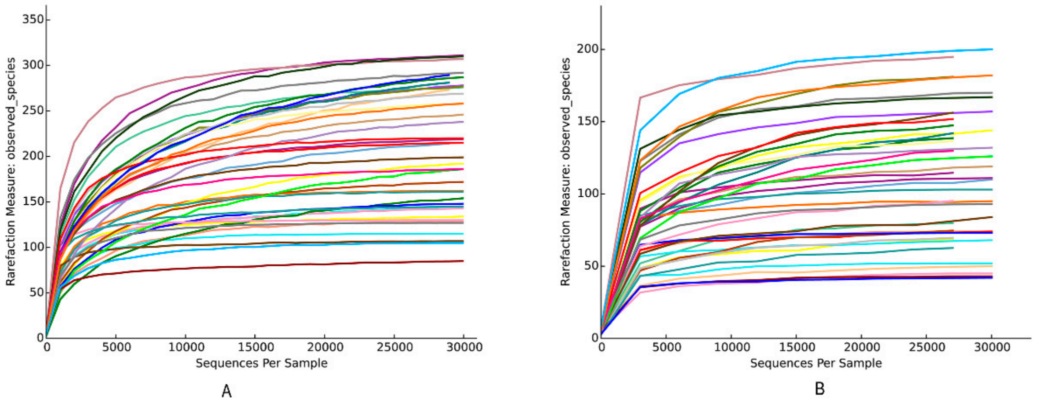

Figure S1. Rarefaction curves of fungal OTU (operational taxonomic unit) richness in the four *Habenaria* species with difference primer pairs. (A) ITS3/ITS4OF and (B) ITS86F/ITS4.

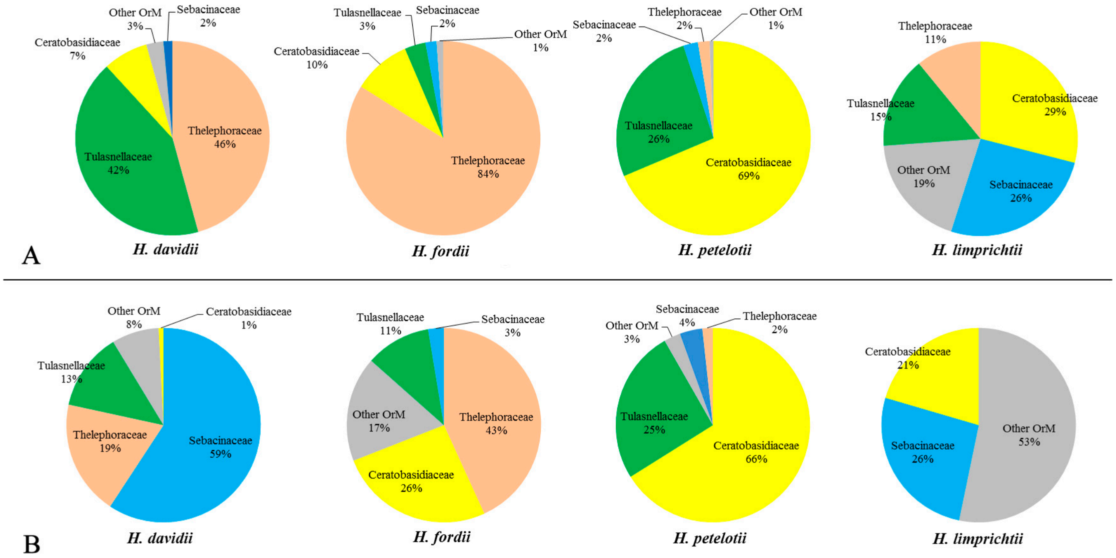

Figure S2. Frequency distribution of identified operational taxonomic units (OTUs) of the four most abundant OMF families (Ceratobasidiaceae, Tulasnellaceae Thelephoraceae and Serendipitaceae) and the other OMF (the other OMF genus described in Table S2) obtained from the roots of the four studied *Habenaria* species using primer pairs (A) ITS3/ITS4OF and (B) ITS86F/ITS4.
